# Discovery of Kinase and Carbonic Anhydrase Dual Inhibitors by Machine Learning Classification and Experiments

**DOI:** 10.3390/ph15020236

**Published:** 2022-02-16

**Authors:** Min-Jeong Kim, Sarita Pandit, Jun-Goo Jee

**Affiliations:** Research Institute of Pharmaceutical Sciences, College of Pharmacy, Kyungpook National University, 80 Daehak-ro, Buk-gu, Daegu 41566, Korea; dns01185@naver.com (M.-J.K.); saritapandit94@gmail.com (S.P.)

**Keywords:** carbonic anhydrase, cheminformatics, docking simulation, dual inhibitor, fingerprint, kinase, machine learning, polypharmacology

## Abstract

A multi-target small molecule modulator is advantageous for treating complicated diseases such as cancers. However, the strategy and application for discovering a multi-target modulator have been less reported. This study presents the dual inhibitors for kinase and carbonic anhydrase (CA) predicted by machine learning (ML) classifiers, and validated by biochemical and biophysical experiments. ML trained by CA I and CA II inhibitor molecular fingerprints predicted candidates from the protein-specific bioactive molecules approved or under clinical trials. For experimental tests, three sulfonamide-containing kinase inhibitors, **5932**, **5946**, and **6046**, were chosen. The enzyme assays with CA I, CA II, CA IX, and CA XII have allowed the quantitative comparison in the molecules’ inhibitory activities. While **6046** inhibited weakly, **5932** and **5946** exhibited potent inhibitions with 100 nM to 1 μM inhibitory constants. The ML screening was extended for finding CAs inhibitors of all known kinase inhibitors. It found XMU-MP-1 as another potent CA inhibitor with an approximate 30 nM inhibitory constant for CA I, CA II, and CA IX. Differential scanning fluorimetry confirmed the direct interaction between CAs and small molecules. Cheminformatics studies, including docking simulation, suggest that each molecule possesses two separate functional moieties: one for interaction with kinases and the other with CAs.

## 1. Introduction

A molecule targeting a single disease-related protein often causes insufficient efficacy, even potent from the viewpoint of target engagement. Therapeutic strategies for treating cancer, neurodegenerative, cardiovascular, and infectious diseases often necessitate multiple target simultaneous modulation due to their complicated origins [1,2,3]. Targeting numerous targets may increase side effects. There is an increasing interest in developing therapeutics that selectively manipulate multiple desired pathways at once [4].

Two approaches are known for simultaneous control. The first method is a mixture of monotherapies, including a combination of drugs with an active ingredient and a single formula comprising multiple active ingredients. The second approach is a single molecule that can modulate several targets. Multi-target-directing molecules are advantageous because of the fewer side effects and toxicities [5,6]. Instead, finding and designing a molecule with multiple functions is not straightforward, necessitating an effective strategy [4,7].

One of the diseases where the multiprotein-targeting modulators are necessary is cancer [8]. Cancer cells have changed signal pathways. The altered pathways are the causes and effects of the disease. Drugs targeting several cancer pathways have been developed. Notably, more than 40 small molecule kinase inhibitors have been approved [9]. Meanwhile, cancer cells require more control of pH. Carbonic anhydrases (CAs) catalyze the production of carbonate ion from carbon dioxide, and mono- and di-carbonate are part of the central buffer systems in our bodies. The increased modulation of pH requires the hyper-functioning CAs. Indeed, the relation between the decreased survival rates and the elevated CA IX and CA XII in triple-negative breast cancer has been reported [10]. Reliance on the role of CAs by cancer cells has made CA a target for cancer therapy. Of accumulated CA inhibitors, dual inhibitors with kinase have been reported [11,12,13]. Nevertheless, the dual inhibitors are limited, and there is no established strategy to find new dual inhibitors.

Accumulating chemical and biological data enables the prediction of small bioactive cellular target molecules. Computational methods have helped to decode off-targets and polypharmacological effects with the data. The similarity ensemble approach (SEA) is a representative example [14,15,16]. Successful SEA applications have been reported for predicting off-targets of FDA-approved drugs and excipients [17,18,19]. Besides the SEA, several computational approaches have been reported [20,21,22,23,24]. Of them, some employ machine learning (ML) [24].

ML is an approach to finding the hidden rule among the accumulated data. Classification, regression, and clustering are three areas applied by ML. If the data are significant, one can apply deep learning using neural networks. Two crucial factors for ML are the features to train and the algorithm. How to select and optimize the pair determines the performance of ML. Popular features to employ ML for small molecule research are molecular fingerprints. Several applications have been reported for discovering new inhibitors against a specific target, including the inhibitor screenings for JAK2 and FGFR4 kinases [25,26]. However, the applications to discover multiple modulators or find off-targets are less known. The coupled experimental validation with the prediction is especially limited.

This study reports the dual inhibitors of kinases and CAs using ML and experiments. The small molecule inhibitors of CA I and CA II were gathered and treated as active. Their physicochemically matched but topologically different decoys were generated for use as inactive molecules. The conversion of each molecule into a molecular fingerprint and the trainings using the ML classifiers followed. Use of an external dataset allowed selection of the best pair of features and classifiers. The selected ML classifier screened the database of experimentally validated protein-specific modulators. The successive biochemical and biophysical experiments confirmed the inhibitory activities of the predicted kinase inhibitors against CAs. The ML classifier was extended for screening all known kinase inhibitors. The cheminformatics, including docking simulation, also provide the chemical features of the dual inhibitors.

## 2. Results

### 2.1. The Pair of ECFP4 Fingerprint and Logistic Regression Classifier Was Selected for Screening

We retrieved the inhibitors for human CA I (UniProt ID: P00915) and CA II (P00918) from BindingDB [27]. Here, molecules smaller than 500 Da and more potent than 500 nM in terms of inhibitory constant (K_i_) or half-maximal inhibitory concentration (IC_50_) were considered. Once clustering the molecules with the cutoff of 0.6 Tanimoto coefficient (TC), the most potent ones in each cluster were representatively chosen as active. This is to minimize the bias in the chemical spaces of the ligands. Then, the DUD-E server [28] generated 40 decoys per inhibitor, which were treated as inactive. No decoy shares similarity higher than 0.6 to inhibitors and 0.8 to each other in TC. The respective numbers of actives (inactives) for CA I and CA II were 167 (6627) and 302 (11,968). We then digitized the SMILES of each chemical with three fingerprints [29,30], extended-connectivity fingerprint 4 (ECFP4), extended-connectivity fingerprint 6 (ECFP6), and molecular access system keys (MACCS), and used them as the features for ML classification. For ML classification, we used seven methods, which are K-Nearest Neighbor (KNN), Naïve Bayesian (NB), Logistic Regression (Logit), Decision Tree (DT), Random Forest (RF), Multi-Layer Perceptron (MLP), and Extreme Gradient Boosting (XGBoost).

Table 1 indicates the result metrics by ML classification with three fingerprints and seven models in two proteins of CA I and CA II (21 cases per protein). The metrics for judging the results by each ML classifier are accuracy (ACC), the area under the curve of receiver operating characteristics curve (AUC), and the Matthews correlation coefficient (MCC). The values of ACC and AUC were comparable with the values approximating 1, which makes them less helpful in discriminating the models. Meanwhile, MCC values differed depending on the combination. MCC is a suited metric reflecting true and false positives and negatives of varying sizes [31]. The abundance of inactive data in screening can bring about substantial false positives when the classification is inaccurate. In this situation, MCC can be the metric for reflecting the reliability of the ML classifier. Overall, the ECFP4 fingerprint exhibited better results than those with ECFP6 and MACCS. Indeed, many publications have used ECFP4 as a fingerprint [14,17,18,19,24,25,32,33]. The models of Logit and MLP revealed comparable and higher MCC values than other methods. Therefore, we focused on the two methods.

Using an external dataset can help choose the better ML model [34]. The discriminating powers in the pairs of ECFP4 and Logit, and ECFP4 and MLP were compared using an external dataset of FDA-approved drugs. The 1318 small molecules registered as “Approved drugs” in the International Union of Basic and Clinical Pharmacology database (IUPHAR) and having a molecular weight smaller than 700 Da were extracted for the validation [35]. The family of diuretic drugs includes CA inhibitors. All the analogs showing the similarity to FDA-approved drugs with TC ≥ 0.6 were excluded from the ligands in the training dataset for a fair comparison. Their corresponding decoys were also omitted from inactive molecules. Logit and MLP classifiers trained with the remaining CA I dataset retrieved seven molecules as common candidates. An extensive literature search confirmed that these seven molecules are direct binders to at least one of the CAs (Table 2 and Figure S1). The CA inhibitors are valdecoxib (IUPHAR ID: **2894**), sulpiride (**5501** and **958**), acetazolamide (**6792**), diclofenamide (**6807**), methazolamide (**6828**), and zonisamide (**7047**). Logit and MLP classifiers trained using CA II dataset predicted six common true positives, which are celecoxib (**2892**), valdecoxib (**2894**), acetazolamide (**6792**), diclofenamide (**6807**), ethoxzolamide (**6814**), and methazolamide (**6828**). These have also been known to directly interact with CAs. When compared to those from Logit, the results from MLP classifiers were worse, predicting more non-binders as candidates both in CA I and CA II cases (Table 2 and Figure S1). Altogether, it was judged that Logit classifier is more suited for our study. Notably, Logit classifier is simpler than MLP in terms of the parameters’ numbers. A simpler model can decrease the concerns on overfitting if the discriminative powers are comparable.

### 2.2. Screening Known Protein-Specific Modulators by ML Classified the Candidate Inhibitors for CA

IUPHAR-registered “Synthetic organics” 6975 molecules smaller than 700 Da were screened using the two paired ECFP4 and Logit classifiers trained with CA I and CA II datasets. IUPHAR database contains only experimentally validated protein-specific modulators, mostly FDA-approved or under clinical trials [35]. CA I and CA II cases led to 42 and 49 candidates with *p* ≥ 0.5, respectively (Table 3 and Figure S2). Of them, 23 molecules are common in CA I and CA II predictions. Their on-targets are various, but the primary targets are CAs in 13 molecules (Table 3). It covers 72% (=13/18) of the total 18 CA inhibitors in the database (Figure S3). Interestingly, the candidates include **4836** (*p* = 0.897, hydrochlorothiazide) and **7197** (0.509, hydroflumethiazide). They are unassigned as CA inhibitors in the IUPHAR database. However, the literature search found that they inhibit CAs [36], demonstrating the performance of our ML-based prediction.

Our proof-of-concept study focused on the kinase inhibitors of unverified CA inhibitor candidates. The literature search identified four potent kinase inhibitors, **5932** (JNJ-7706621) [37], **5946** (CDK1/2 inhibitor III) [37], **8146** [38], and **6046** [39]. The molecules’ primary targets are cyclin-dependent kinases (CDKs) and mammalian sterile 20-like kinase 3 (MST3) for **5932** (IC_50_ for CDK2: 2 nM) and **5946** (IC_50_ for CDK2: 0.5 nM), TXK tyrosine kinase for **8146** (IC_50_ for TXK: 0.4 nM), and spleen associated tyrosine kinase (SYK) for **6046** (IC_50_ for SYK: 15.8 nM). All target proteins are related to cancers. We chose **5932**, **5946**, and **6046** for experimental tests considering commercial availability. A literature search revealed that pazopanib (IUPHAR ID: **5698**) is inhibitory in both CAs and kinases such as CSF1R [13], although ML could not list it as a candidate. Acetazolamide (**6792**) and pazopanib were used as positive controls. There are a number of notable points. First, active molecules for training did not include **5932**, **5946**, and **6046**. The closest compounds of **5932**, **5946**, and **6046** in the training data were ZINC28389402, ZINC28389402, and ZINC13804313 with the TC values of 0.460, and 0.407, and 0.270, respectively (Table 3). Second, all the molecules have a sulfonamide moiety needed to chelate with the zinc atom in the catalytic core. Third, **5932** was not listed by the CA II model, despite the shared similarity to **5946** with a TC of 0.685.

### 2.3. Two Candidates, **5932** and **5946**, Were Highly Inhibitory for CAs

**Figure 1:**
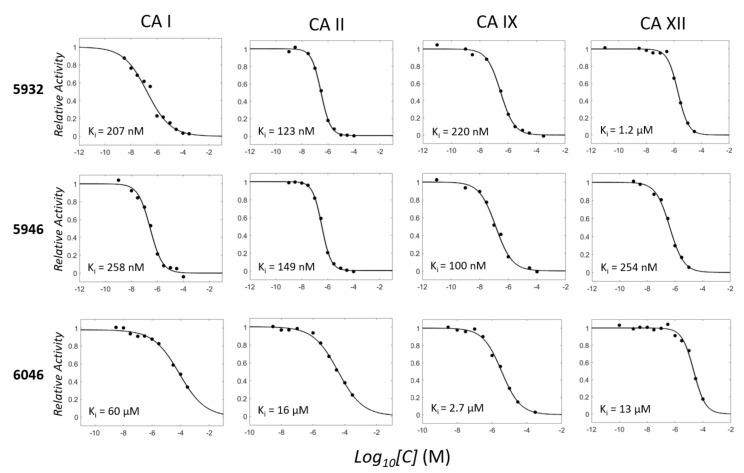
Concentration-dependent inhibitory profiles. Enzyme activities are scaled to have a relative value in the range of 0–1 on the y-axis. The enzyme activity in the absence of an inhibitor is expressed as one. The molar concentrations in each small molecule ([*C*]) are expressed as *Log_10_[C]* on the x-axis. The K_i_ values that are converted from the fitted IC_50_ values are written with units.

**Figure 2:**
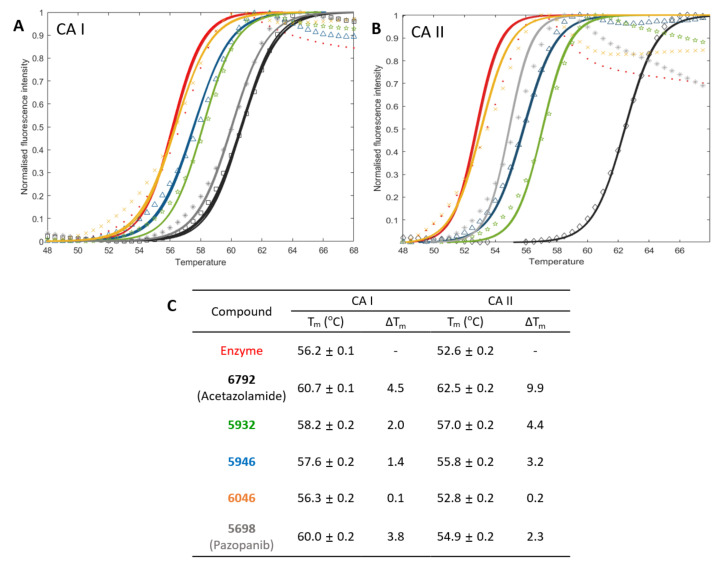
Profiles of differential scanning fluorimetry. Temperature-dependent changes of fluorescence from SYPRO^®^ Orange in the mixture of protein and inhibitor are fitted for calculating the mid-temperature (T_m_) of denaturation. Fluorescence intensities are normalized with relative fluorescence units in the range of 40 °C–68 °C. Markers and lines indicate the raw and fitted data in each case, respectively. The DSF profiles (**A**,**B**) and the name (**C**) in each compound are prepared to have identical colors. Three independent experiments were repeated for calculating means and standard deviations.

Two candidates, **5932** and **5946**, exhibited potent inhibition against CAs, but the inhibition by **6046** was moderate (Figure 1 and Figure S3, and Table 4). The K_i_ values for **5932**, **5946**, and **6046** were 123 nM, 149 nM, and 16 μM in CA II, whereas they were 207 nM, 258 nM, and 60 μM in CA I, respectively. We also quantified the inhibition toward two anticancer therapeutic targets, CA IX and CA XII. The K_i_ values for CA IX were 220 nM, 100 nM, and 2.7 μM for **5932**, **5946**, and **6046**, while CA XII revealed 1.2 μM, 254 nM, and 13 μM values. Except for CA XII, **5932** and **5946** showed similar inhibitory constants. It is noteworthy that all the reaction mixtures contain 0.01% Triton X-100 to avoid the aggregation-based enzyme inhibition by small molecules [40,41,42,43]. The direct bindings of **5932**, **5946**, and **6046** to CA I and CA II were checked using differential scanning fluorimetry (DSF) (Figure 2). The measured ΔT_m_ from the apo CA I were in the descending order of **6792** (4.5 degrees), **5698** (3.8), **5932** (2.0), **5946** (1.3), and **6046** (0.1). The degrees of ΔT_m_ were more distinct in CA II. The order was **6792** (9.9), **5632** (4.4), **5946** (3.2), **5698** (2.3), and **6046** (0.2). All shifted beyond 3 × standard deviation from the reference except for **6046**, reflecting the significance of the change and thus direct interactions. The data qualitatively agree with enzyme assay, supporting the consistency in the orthogonal experimental confirmation.

### 2.4. Cheminformatics Demonstrated Unique Features Compared to Other CA Inhibitors

**Figure 3:**
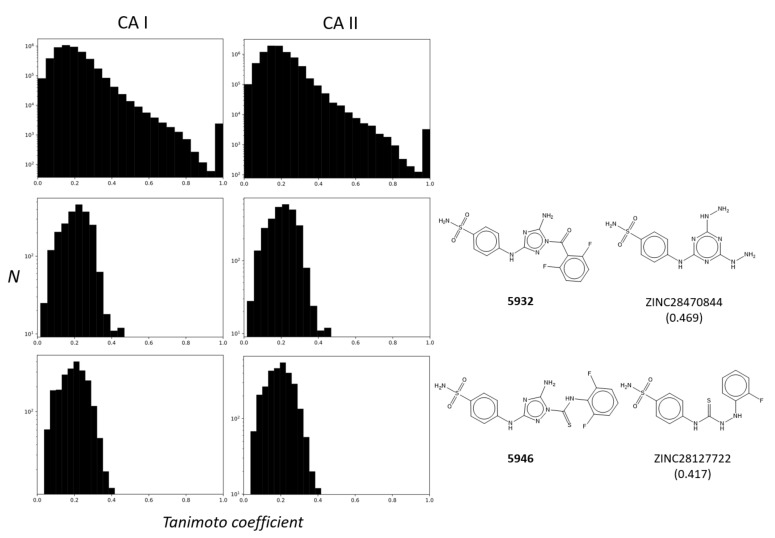
Cheminformatics using similarity ensemble approach. Distributions of TCs between the pairs in a set of known CA I and CA II inhibitors (**upper**) are expressed using histogram as references. TCs of two new inhibitors, **5932** (**middle**) and **5946** (**bottom**), and the known CA I and CA II inhibitors, are computed for comparison. The closest known CA inhibitors registered in BindingDB [25] are drawn with the TCs in the parentheses.

Are the chemical scaffolds of **5932** and **5946** unique compared to the other known CA inhibitors? To answer this question, we inspected the distribution of TCs between **5932** and **5946,** and other known inhibitors in CA I and CA II. In this SEA, the distribution’s biased shape toward one on the x-axis reflects the shared chemical similarities between two groups of inhibitors [14]. Compared to the cases of CA I and CA II inhibitors, the distributions between **5932** and **5946** and the known inhibitors were constrained, with values mostly smaller than 0.4 (Figure 3). The closest similarities were found in ZINC28470844 with a TC of 0.469 for **5932** and ZINC28127722 with 0.417 for **5946**. Indeed, the apparent similarities are limited, except for benzenesulfonamide moiety (Figure 3). This data can qualitatively support the scaffolds’ novelty in **5932** and **5946**.

### 2.5. Docking Simulation Predicted the Binding Modes of the Dual Inhibitors

To obtain detailed information at the atomic level, we performed a docking simulation. Direct inhibitors targeting metalloenzymes usually require a metal-binding group (MBG) to interact with the catalytic metals [44]. Sulfonamide is the representative MBG for CAs. Considering the shared sulfonamide, one can infer that **5932** and **5946** interact with CA with the same moiety. The protein database contained 58 coordinates of CA I from 33 crystal structures in the absence or presence of inhibitors. Complex structures with sulfonamide-containing inhibitors have shown a lack of a proton in the amide. It makes the moiety have the net charge of −1, enabling the chelation between the nitrogen and the zinc metal. The proton’s exclusion was approximated by setting pH as 14.0 in the ligand preparation.

The pair of Glide-SP [45,46] and 4WUP-B (4WUP Chain B) of CA I [47] was chosen as a docking engine and a template coordinate, respectively. Two key factors considered for judging structure-based docking are pose reproduction and early enrichment of true positives over false positives. We first tested which software reproduced the crystal poses most faithfully of DOCK3.7 [48,49], Fred [50,51], and Glide-SP [45,46]. Once aligning 58 coordinates with an identical direction, 24 inhibitors from the crystal structures were docked into the structures using each software. The runs using Glide-SP showed the best results with reasonable energies and geometries. Then, the total of 960 decoys (40 per ligand, 960 = 24 × 40) was prepared with the identical method applied for ML. The docking simulations using Glide-SP into each coordinate followed with a set of inhibitors and decoys. The early enrichment of true positives in the docking with 4WUP-B was shown as the best with AUC and LogAUC [48] values of 86.3 and 20.8 (Figure S4). Note that the preparation of small molecules by setting pH as 14.0 is indispensable to obtain the desirable profiles.

**Figure 4:**
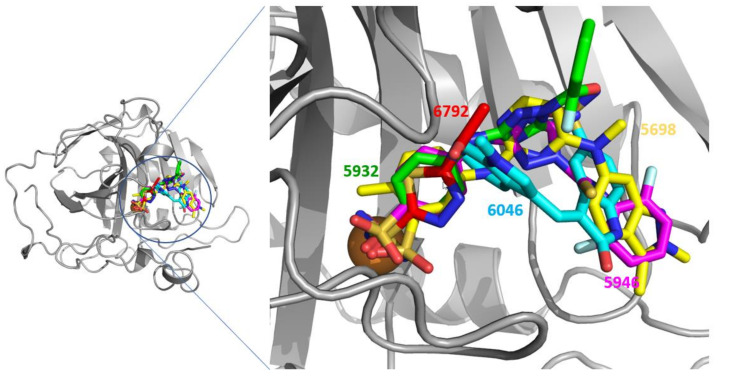
Docking simulation. The docked poses between CA I and the molecules in this study are prepared using Glide and the coordinate of 4WUP-B. The molecules in red, green, magenta, cyan, and yellow indicate **6792** (acetazolamide), **5932**, **5946**, **6046**, and **5698** (pazopanib), respectively. The sphere of brown is the zinc ion. The ligands for docking were prepared setting pH as 14 using LigPrep of Schrödinger package.

Docked poses indicated that **6792**, **5932**, **5946**, and **5698** contacted the zinc atoms through the sulfonamide. Particularly, **6792** had an identical pose to that of the crystal structure. However, **6046** displayed entirely different poses, not contacting the zinc (Figure 4). These data are consistent with the experimental observation that **6046** is less inhibitory for CAs. It shows that the presence of the sulfonamide moiety is not a sufficient condition for inhibiting CAs. Both the existence of MBG and the geometrical fitness of other parts are equally essential.

Discriminating the functional moieties for recognizing CAs and kinases is valuable. Comparing the docked poses of **5932** and **5946** in CA I to those in crystal kinase structures would allow a detailed understanding of the multiple and specific inhibition by **5932** and **5946**. The protein databank includes 13 crystal structures with **5932** as a ligand named SKE (http://www.rcsb.org/ligand/SKE, accessed on 1 April 2021). Eleven belong to kinase or pseudokinase domains (PDB IDs: 3AMA, 4QMU, 5DPV, 5DR6, 5DR9, 5DT0, 5OBR, 5USY, 5USZ, 5WIN, and 6DRW), and two cases are spastin AAA domain (6P10 and 6P11). The kinases are MST3 and Aurora A kinases. Additionally, 10 crystal structures with **5946** are found to have the name of DKI (http://www.rcsb.org/ligand/DKI, accessed on 1 April 2021). The PDB IDs are 2CHL, 2J51, 2JFL, 2W4O, 2WU6, 3HMI, 3LJ1, 4AAA, 4FZF, and 4QMP. All proteins belong to the kinase family, including ABL2, casein kinase, CLK3, and MST3. Notably, the kinase structures complexed with **5932** and **5946** have a DFGin conformation at the kinase activation loop [52].

**Figure 5:**
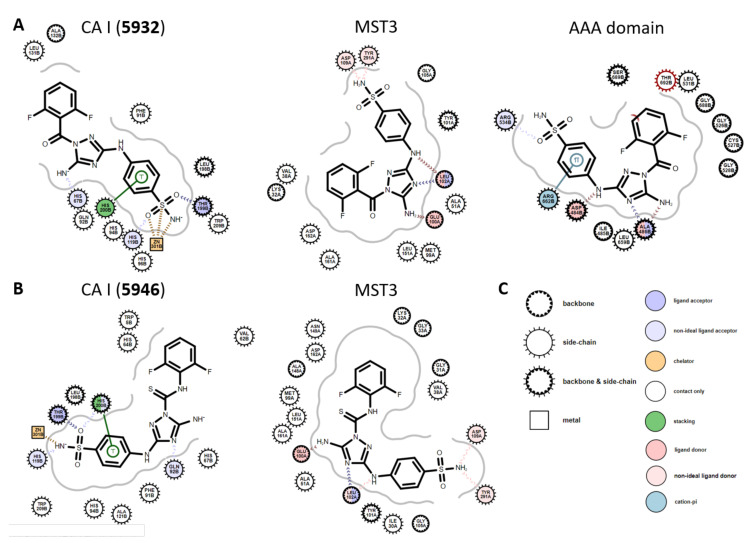
Comparison of poses in carbonic anhydrase and other proteins. (**A**) 2D diagrams of intermolecular interactions of **5932** with CA I (docked model), MST3 kinase (PDB ID: 4QMU), and AAA domain (6P10). (**B**) 2D diagrams of intermolecular interactions of **5946** with CA I (docked model) and MST3 kinase (4QMP). (**C**) Residue and interaction styles used in (**A**,**B**) are defined with the shapes and colors of circles according to the OpenEye Grapheme Toolkit.

In MST3 (4QMU) and AAA domain (6P10) structures, the sulfonamides of **5932** exist on the solvent-accessible surface, forming contacts with the side chains of aspartate and tyrosine. This interaction seems not to be the key contact compared with other kinase inhibitors. AAA domain additionally uses arginine to interact with the aromatic through intermolecular π-cation contact. Meanwhile, the docked models indicate that the sulfonamide mediates the chelation with the catalytic zinc atom in the core of CAs. The existence of the next aromatic group to sulfonamide is also important by forming intermolecular π-π stacking with histidine. Both kinase and AAA domains use amino-triazole for intermolecular hydrogen bonds (Figure 5A). The poses of **5946** were similar to those of **5932** in CA I and MST3, respectively. The benzenesulfonamide plays a crucial role in intermolecular interaction with CA I. Triazole diamine is more vital for interacting with kinase (Figure 5B).

### 2.6. *ML Screening of the Known Kinase Inhibitors Led to Discovering a New Potent CA Inhibitor, XMU-MP-1*

**Figure 6:**
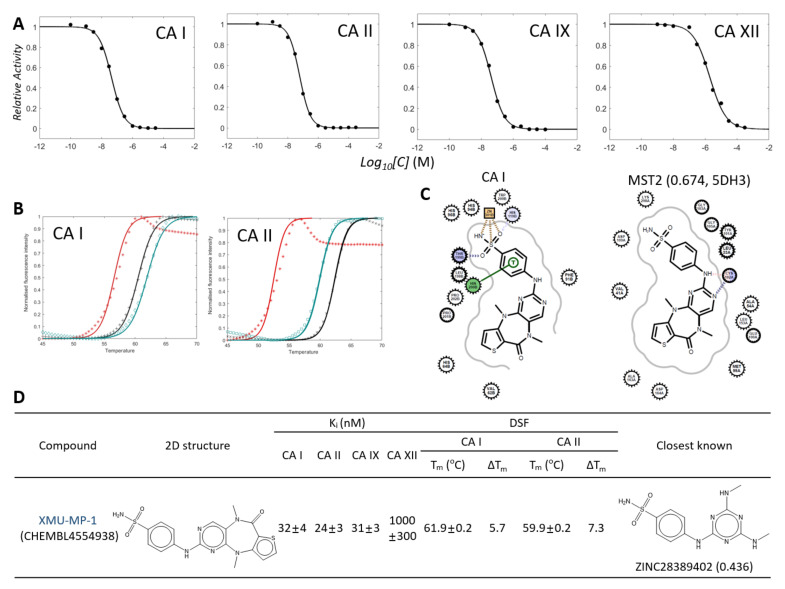
Inhibition of carbonic anhydrase by XMU-MP-1. Profiles and uncertainty analyses of enzyme inhibition by XMU-MP-1 are prepared identically to Figure 1 and Figure 2 and Table 4. (**A**) Enzyme inhibitory profiles. (**B**) Temperature-dependent fluorescence changes by DSF. Red, black, and navy lines correspond to those without inhibitor, with acetazolamide, and with XMU-MP-1, respectively. (**C**) 2D diagrams of intermolecular interactions of XMU-MP-1 in docked with CA I and in MST2 kinase structure. The values in the parentheses are probability by ML classifier and PDB ID. (**D**) Chemical structure, the values of K_i_ in enzyme activity, and T_m_ in DSF with XMU-MP-1 are presented. The closest molecule to XMU-MP-1 of the known CA I inhibitors is drawn with the TC in the parenthesis.

A meaningful question is whether our approach is extensible to a larger database for finding dual kinase and CA inhibitors. We extracted 35,455 kinase inhibitors of 437 kinases from the ChEMBL database [53]. The molecules smaller than 450 Da and more potent than 500 nM in K_i_ or IC_50_ were considered. We then applied the ML classifier of the CA I model. It retrieved 192 kinase inhibitors with *p* ≥ 0.6 as candidates for CA inhibition. Most molecules have a sulfonamide moiety, but those without the moiety also exist (Figure S5). Interestingly, the candidates include eight molecules whose complex structures with kinases are available. Besides **5932** (CHEMBL191003) and **5946** (CHEMBL261720), the other six molecules were CHEMBL2377825 (*p* = 0.965, PDB ligand name: 106, and PDB ID: 1FVT), CHEMBL231950 (0.923, X64, and 3QXP), CHEMBL233149 (0.893, C85, and 2UZD), CHEMBL319467 (0.847, 4SP, and 1H1S/5M57/6BSS), CHEMBL585367 (0.752, ZZF, and 2WOU), and CHEMBL4554938 (0.674, 5BS, and 5DH3) (Figure 6 and Figure S6). Notably, the IUPHAR database does not include these six molecules.

Of these molecules, considering the commercial availability and feasibility of a detailed comparison, we selected CHEMBL4554938 (XMU-MP-1) and tested its inhibition for CAs. XMU-MP-1 was developed to pharmacologically inhibit MST1/2 kinases [54]. The reported IC_50_ values for MST1 and MST2 are 71 and 31 nM. Remarkably, XMU-MP-1 was a potent inhibitor for CA I, CA II, and CA IX with the K_i_ values of 32, 24, and 31 nM, respectively (Figure 6A,D). The K_i_ for CA XII was 1 μM. DSF also verified the direct interaction between XMU-MP-1 and CA I and CA II (Figure 6B,D). Docking simulation and crystal structure indicated that the intermolecular contacts found in the bound poses of XMU-MP-1 to CA I and kinase are similar to **5932** and **5946** cases (Figure 6C). While the benzenesulfonamide is crucial for the interaction with the zinc atom of CA I, the key interaction with the hinge region of MST2 kinase uses 2-aminopyrimidine of XMU-MP-1 [54]. The activation loop of the kinase structure with XMU-MP-1 has a DFGin conformation too [52]. The chemical similarity to the known CA inhibitors is limited in the sulfonamide moiety. The closest known inhibitor is ZINC28389402 with a TC of 0.436 (Figure 6D).

## 3. Discussion

Rapidly accumulating chemical and biological data are expected to accelerate more accurate computational screens. However, imperfectness in the algorithm and the training dataset brings about vast false positives in computer-assisted screens [55,56], necessitating experimental confirmation. Quantitative prediction using ML regression is more challenging than classification. The optimization of ML regression relies on the loss function, expressed mainly as the mean squared error. Comparing the loss functions with k-fold cross-validation is the way to select the best model. By contrast, ML binary classification allows the use of various metrics. It is advantageous for judging several models simultaneously. In addition, ML classification is less sensitive to erroneous data than ML regression. A few errors can significantly influence the performance of ML regression. Our data showed that MCC [31] is the discriminative metric for selecting the more proper ML classification model, whereas ACC discriminates little. We even employed another approach to compensate for the imperfectness of ML, the validation using an external dataset. When the number of true positives is small, a direct comparison of true positives would be straightforward for selecting the best approach. Notably, our approach is readily extensible for discovering other types of dual inhibitors as long as the information for inhibitors is available.

A comprehensive understanding of the polypharmacology of a small molecule is crucial for developing selective drugs and for studying signaling pathways mediated through the target protein. Unidentified targets of a bioactive molecule may impact the interpretation made under the assumption of a single on-target effect. XMU-MP-1 is the chemical probe widely being used to study the Hippo pathway. Several publications have described the cellular roles of MST1/2 in the Hippo pathway with XMU-MP-1, assuming the molecule can pharmacologically inhibit the function of MST1/2 [54,57,58,59,60,61,62,63]. Our study showed that XMU-MP-1 is a comparably potent inhibitor for CA I, CA II, and CA IX to MST1/2. This raises the possibility that CAs inhibition influences the experimental observations. Indeed, the linkage between CAs and TEAD-YAP/TAZ in the Hippo pathway has been reported [64]. Similarly, the interpretation of cellular studies using 5932 and 5946 may require caution on off-target effects through CA inhibition.

For maximizing the therapeutic effects of kinase and CA dual inhibitors as anticancer agents, one must consider kinase type-specific and CA isoform-specific dual inhibitors. Our proof-of-concept study has mainly focused on the feasibility of the combined use of ML binary classification and experiments to discover dual inhibitors. The massive experimental tests for selective kinase and CA dual inhibitors are beyond the scope of this study. Nevertheless, our data provide meaningful quantitative information. XMU-MP-1 is much weaker in inhibiting CA XII by approximately 30-fold, and **5946** shows more potent inhibition for CA IX. Moreover, the 192 candidates (Figure S5) can hopefully trigger the research on selective kinase and CA dual inhibitors, particularly to find more selective CA IX and CA XII inhibitors.

Barelier et al. classified the recognition of identical ligands in unrelated proteins into three interactions: identical, similar, and unshared [65]. The dual inhibition by **5932**, **5946**, and XMU-MP-1 for kinases and CAs belongs to the unshared recognition. The key contacts occur in the nonoverlapping parts of **5932**, **5946**, and XMU-MP-1. A synthetic modification for modulating polypharmacological effects such as selectivity might be more straightforward in these cases. Our docking protocol will help prepare the accurate poses of designed molecules.

Our approach minimally optimized the parameters of ML classification. Docking simulation was applied not for screening but for understanding the binding modes of active molecules. The physics-based docking screen can work synergistically with the informatics-based ML classification, particularly for screening the ultra-large library comprising more than several billion molecules [15,16]. ML is faster than conventional docking by several hundred-fold and suited for primary crude screening. Subsequent docking can decrease the ML-predicted candidates into manageable numbers for experimental tests. Of note, our optimized docking protocol for CA has the potential to rule out the weak inhibitor, **6046**, by checking the docked pose. The co-use of an optimized ML and docking screen would be a research area deserving further study [15,16]. Our data will be a notable addition to this direction.

## 4. Materials and Methods

For the ML database, BindingDB [27] was used to extract inhibitors for CA I and CA II. The method of DUD-E server [28] was employed to generate physicochemically matched but topologically different decoys per ligand. The small molecules registered as “Approved drugs” and “Synthetic organics” in IUPHAR and with the molecular weight smaller than 700 were extracted for validation and screening [35], respectively.

The ML classification models were prepared using the Scikit-learn (version 0.22) library implemented in Python (version 3.7) by combining three fingerprints and seven models with default parameters. Setting the radii as 2 and 3 Å of Morgan circular fingerprints in RDKit (ver. 2020.03.3, http://www.rdkit.org, accessed on 1 April 2021) approximated ECFP4 and ECFP6 fingerprints comprising 1024 bits. MACCS fingerprint judges whether the predefined 166 patterns exist or not in a molecule, comprising 166 bits. Each bit has the values of zero or one dependent on the absence or presence of a functional moiety. The seven classifier models in test were K-Nearest Neighbor (KNN), Naïve Bayesian (NB), Logistic Regression (Logit), Decision Tree (DT), Random Forest (RF), Multi-Layer Perceptron (MLP), and Extreme Gradient Boosting (XGBoost). Once randomly dividing the data into five blocks of equal sizes, five-fold cross-validation followed with five runs. Here, training and validation parts in a run consisted of four and one parts. Average and standard deviation was calculated for each metric. The final model for screening database was prepared using all the input data.

Binary classification of ML has the four resulting classes: true positive (TP), true negative (TN), false positive (FP), and false negative (FN). Three metrics for quantifying the qualities of the ML model are accuracy (ACC), the area under the curve (AUC) of receiver operating characteristic curve (ROC), and Matthews correlation coefficient (MCC). ACC and MCC are defined as follows:

$$\begin{array}{l} \mathrm{ACC}=\frac{\mathrm{TP}+\mathrm{TN}}{\mathrm{TP}+\mathrm{TN}+\mathrm{FP}+\mathrm{FN}}\\ \mathrm{MCC}=\frac{\mathrm{TP}\times \mathrm{TN}-\mathrm{FP}\times \mathrm{FN}}{\sqrt{\left(\mathrm{TP}+\mathrm{FP})(\mathrm{TP}+\mathrm{FN})(\mathrm{TN}+\mathrm{FP})(\mathrm{TN}+\mathrm{FN}\right)}} \end{array}$$

ROC is a two-dimensional expression of TP and FP ratios in the respective y- and x-axes. AUC is defined as the area in the ROC. AUC represents the enrichment of TP over FP.

All the reagents used for the biochemical experiments for enzyme inhibitions were bought from Cayman (Ann Harbor, MI, USA), Sigma–Aldrich (St. Louis, MO, USA), Sino Biological (Beijing, China), or Tokyo Chemical Industry (Tokyo, Japan). Inhibitory activities of small molecules on CAs were measured using 4-nitrophenyl acetate as a substrate. The conversion from the substrate into *p*-nitrophenol by CAs led to the optical density change at 405 nm. The changes were measured at 96-well microplates using Epoch2 from BioTek (Winooski, VT, USA). The slope in a time-dependent measurement was calculated by linear regression and interpreted as the quantified activity. Each 200 μL reaction solution comprised 5 nM enzyme and 1 mM substrate buffered using phosphate-buffered saline containing 5% dimethyl sulfoxide and 0.01% (*w*/*v*) Triton X-100 under the absence or the presence of inhibitor. All reactions were performed at 37 °C. The substrate-only solution was used as a control to consider autohydrolysis of the substrate. The value of IC_50_ was converted into K_i_ using the Cheng–Prusoff equation [66].

The biophysical experiments involved the binding of small molecules to CA I or CA II, which were quantified using the T_m_ of DSF. The profile was generated by CFX-connect reverse transcription-polymerase chain reaction system (BioRad, Hercules, CA, USA). The solution contained 5× SYPRO^®^ Orange and 0.5 µM recombinant human CA I or CA II in the absence or presence of 200 µM inhibitor. By gradually elevating the solution’s temperature 30 °C–90 °C with a ramping rate of 1 °C/min, the fluorescence signals of the dye at 610 nm were measured upon excitation at 492 nm. The patterns were nonlinearly fitted to the Boltzmann equation to calculate T_m_ [67]. Only data in the range of 40 °C–68 °C were used for the calculation. Nonlinear curve fittings for K_i_ in enzyme assay and T_m_ in DSF were performed using MATLAB (MathWorks, Natick, MA, USA).

For the cheminformatics and docking simulation, RDKit was employed to calculate the pairwise TCs and to perform SEA using ECFP4 as a molecular fingerprint. For comparing the distributions in TCs, the data from ZINC15 [68] and BindingDB [27] were used. All the molecules more potent than 10 μM in K_i_ or IC_50_ and smaller than 500 Da were selected. Kinase inhibitors were extracted from the ChEMBL database [53]. DOCK3.7 [48,49], Fred from OpenEye (Santa Fe, NM, USA) [50,51], and Glide-SP from Schrödinger (New York, NY, USA) [45,46] were used as engines for docking with the default parameter setting. All the SMILES of ligands were converted into 3D coordinates considering tautomerization and electrostatic charge using LigPrep and Epik from Schrödinger. An in-house script, Automated pLatform for Integrative Structure-based DOCKing (ALIS-DOCK) automated the procedure [69,70]. OpenEye Grapheme was used to analyze the intermolecular interaction in the protein and ligand complexes in docking and PDB-deposited structures.

## Figures and Tables

**Table 1:** The metrics of machine learning classifiers with fingerprints.

CA I	ECFP4			ECFP6			MACCS		
	ACC	AUC	MCC	ACC	AUC	MCC	ACC	AUC	MCC
KNN	0.970	0.950	0.632	0.952	0.932	0.532	0.984	0.916	0.720
NB	0.979	0.697	0.483	0.964	0.660	0.291	0.812	0.868	0.284
Logit	0.994	0.994	0.863	0.993	0.994	0.849	0.989	0.988	0.771
DT	0.988	0.889	0.757	0.985	0.858	0.703	0.985	0.864	0.703
RF	0.992	0.988	0.826	0.990	0.985	0.782	0.990	0.988	0.771
MLP	0.995	0.993	0.883	0.993	0.990	0.843	0.990	0.991	0.794
XGBoost	0.992	0.977	0.832	0.990	0.985	0.797	0.991	0.994	0.815
CA II	**ECFP4**			**ECFP6**			**MACCS**		
	**ACC**	**AUC**	**MCC**	**ACC**	**AUC**	**MCC**	**ACC**	**AUC**	**MCC**
KNN	0.981	0.973	0.734	0.972	0.965	0.659	0.984	0.929	0.739
NB	0.932	0.779	0.331	0.872	0.779	0.268	0.840	0.900	0.321
Logit	0.994	0.998	0.878	0.994	0.998	0.869	0.990	0.993	0.793
DT	0.989	0.899	0.777	0.988	0.868	0.747	0.986	0.862	0.716
RF	0.992	0.997	0.831	0.991	0.996	0.801	0.992	0.995	0.815
MLP	0.994	0.996	0.872	0.993	0.995	0.848	0.991	0.996	0.812
XGBoost	0.992	0.994	0.836	0.992	0.995	0.839	0.990	0.995	0.797

The input dataset comprises 167 actives and 6627 inactive molecules for CA I, and 302 and 11,968 for CA II. The bits for ECFP4, ECFP6, and MACCS fingerprints are 1024, 1024, and 166, respectively. In ML classifiers, KNN stands for K-Nearest Neighbor, NB for Naïve Bayesian, Logit for Logistic Regression, DT for Decision Tree, RF for Random Forest, MLP for Multi-Layer Perceptron, and XGBoost for Extreme Gradient Boosting. The metrics are accuracy (ACC), the area under the curve (AUC) of the receiver operating characteristic curve, and Matthews correlation coefficient (MCC). The values through five-fold cross-validation are averaged in each.

**Table 2:** Validation of machine learning classifiers using the approved drug database.

Protein	Classifier	TP1	TP2	FP	Sum
CA I	Logit	7		2	9
	MLP	7	1	4	12
CA II	Logit	6		5	11
	MLP	6		8	14

True positives 1 (TP1) indicates the known CA targeting drugs predicted both by Logit and MLP classifiers with ECFP4 fingerprint. True positives 2 (TP2) means additional CA-targeting drugs by MLP. The molecules of false positives (FP) are those unrelated to CA inhibitors by the literature search. These data are prepared by ML classifiers trained with other datasets that exclude the actives similar (TC ≥ 0.6) to the approved drugs and their corresponding inactive molecules.

**Table 3:** Candidates from the screen of the known bioactive molecules.

CA I (19)				CA I and CA II (23)				CA II (26)			
ID	Prob	Max TC	ZINC ID	ID	Prob	Max TC	ZINC ID	ID	Prob	Max TC	ZINC ID
3463	0.982	0.394	13800463	**6920**	0.999	0.737	28526472	9753	0.935	0.475	13780724
10070	0.935	0.274	28526472	**6814**	0.995	1.000	56721	3962	0.931	0.290	13612907
4840	0.900	0.288	13650488	**6828**	0.994	1.000	8415468	3986	0.928	0.464	64527862
**7047**	0.838	0.283	58569258	**7046**	0.992	0.422	184018	10483	0.910	0.264	34717999
1376	0.834	0.342	1099	4839	0.990	0.404	13800448	10664	0.903	0.353	87722919
10175	0.801	0.426	131170	**6792**	0.979	1.000	3813042	4836	0.897	0.700	34799864
8821	0.762	0.227	13650488	10017	0.978	0.270	28388514	4742	0.850	0.328	27638369
980	0.751	0.328	22198192	**5946**	0.959	0.407	28389402	10242	0.785	0.474	95586265
9092	0.740	0.192	2101	**10149**	0.948	0.639	84759371	11205	0.776	0.241	64526424
932	0.734	0.283	595377	**8146**	0.912	0.413	13800465	7289	0.757	0.340	13800448
4702	0.724	0.464	1099	2894	0.909	0.435	58569258	8316	0.714	0.375	40917210
960	0.685	0.324	95591272	**6807**	0.905	0.405	13800446	4055	0.680	0.341	26387397
958	0.685	0.324	95591272	**6810**	0.884	1.000	1530622	11201	0.641	0.227	64526424
5501	0.685	0.324	95591272	**6849**	0.838	0.245	34717916	4357	0.623	0.367	27636999
10321	0.589	0.391	84670597	9513	0.830	0.361	84670374	6574	0.599	0.195	13472881
8378	0.548	0.244	1099	7028	0.817	0.380	84652324	4835	0.585	0.360	34799864
**5932**	0.546	0.460	28389402	4837	0.800	0.317	84670374	9129	0.583	0.243	64526424
7409	0.535	0.408	16525334	**6797**	0.722	0.542	1530622	11174	0.580	0.404	27635960
1394	0.509	0.239	5159179	**10433**	0.694	0.541	13829485	10693	0.578	0.406	27741075
				4635	0.661	0.212	13800446	7893	0.572	0.471	16525334
				4084	0.661	0.212	13800446	**2892**	0.571	0.509	95586265
				11220	0.601	0.250	26387397	6514	0.570	0.357	84669523
				7125	0.584	0.318	28349861	**6046**	0.565	0.270	13804313
								7870	0.562	0.425	131170
								7197	0.509	0.690	34799864
								6648	0.507	0.273	27644927

Logistic Regression classification models for CA I and CA II screened IUPHAR “Synthetic organics” comprising 6975 molecules. The candidates are arranged into three criteria: CA I only (the number of candidates: 19), both CA I and CA II (23), and CA II only (26). The ID in IUPHAR, the probability by Logistic Regression classifier, and TC (Max TC) and the name (ZINC ID) of the closest molecule in the training dataset are presented. Those colored in red are known CA inhibitors. Known kinase inhibitors are colored in gray.

**Table 4:** Quantified compound activities.

Compound	2D Structure	CA I	CA II	CA IX	CA XII
**6792** (Acetazolamide)	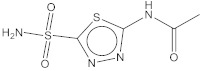	197 ± 18	3 ± 1	22 ± 5	23 ± 10
**5932**	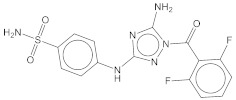	207 ± 66	123 ± 16	220 ± 32	1.2 ± 0.2 (µM)
**5946**	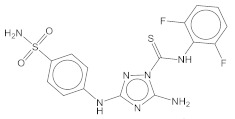	258 ± 58	149 ± 21	100 ± 28	254 ± 40
**6046**	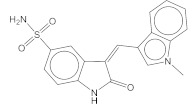	60 ± 9 (µM)	16 ± 5 (µM)	2.7 ± 0.3 (µM)	13 ± 4 (µM)
**5698** (Pazopanib)	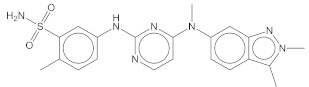	67 ± 10	242 ± 32	1.7 ± 0.5 (µM)	0.8 ± 0.3 (µM)

Enzyme inhibitory activities against CAs by compounds are measured and quantified under a series of small molecule concentrations. The values are inhibitory constant, K_i_. **6792** (acetazolamide) and **5698** (pazopanib) are known CA inhibitors and positive controls in this study. Except for those explicitly labeled, the other values have units of nM. The value following ± is standard deviation that Monte-Carlo simulation of 100 cycles calculates assuming at least 5% uncertainty in experimental data.

## Data Availability

Data is contained within the article.

## Supplementary Materials

The following are available online at https://www.mdpi.com/article/10.3390/ph15020236/s1, Figure S1: CA inhibitor candidates of FDA-approved small molecules by ML classifiers, Figure S2: CA inhibitor candidates from IUPHAR-registered small molecules by ML classifiers, Figure S3: Concentration-dependent inhibitory profiles of positive controls, Figure S4: Data in the pre-docking stage, Figure S5: ML-derived CA inhibitor candidates of known kinase inhibitors, Figure S6: CA inhibitor candidates reported as complex structures with kinases. References [35,45,46,48,53] are cited in the supplementary materials.
