# Supplementary material for: Discovery of Kinase and Carbonic Anhydrase Dual Inhibitors by Machine Learning Classification and Experiments
Source: Pharmaceuticals (Basel). 2022 Feb 16;15(2):236. doi: 10.3390/ph15020236 (PMC8875555; doi:10.3390/ph15020236)
Supplement: Supplementary file 1 [file pharmaceuticals-15-00236-s001.zip › pharmaceuticals-1536458-supplementary.pdf]

Supplementary materials

Discovery of Kinase and Carbonic Anhydrase Dual Inhibitors by Machine Learning Classification and Experiments

Min-Jeong Kim †, Sarita Pandit † and Jun-Goo Jee *

Research Institute of Pharmaceutical Sciences, College of Pharmacy, Kyungpook National University, 80 Daehak-ro, Buk-gu, Daegu 41566, Republic of Korea

* Correspondence: jjee@knu.ac.kr; Tel.: +82-53-950-8568
† These authors contributed equally to this work.

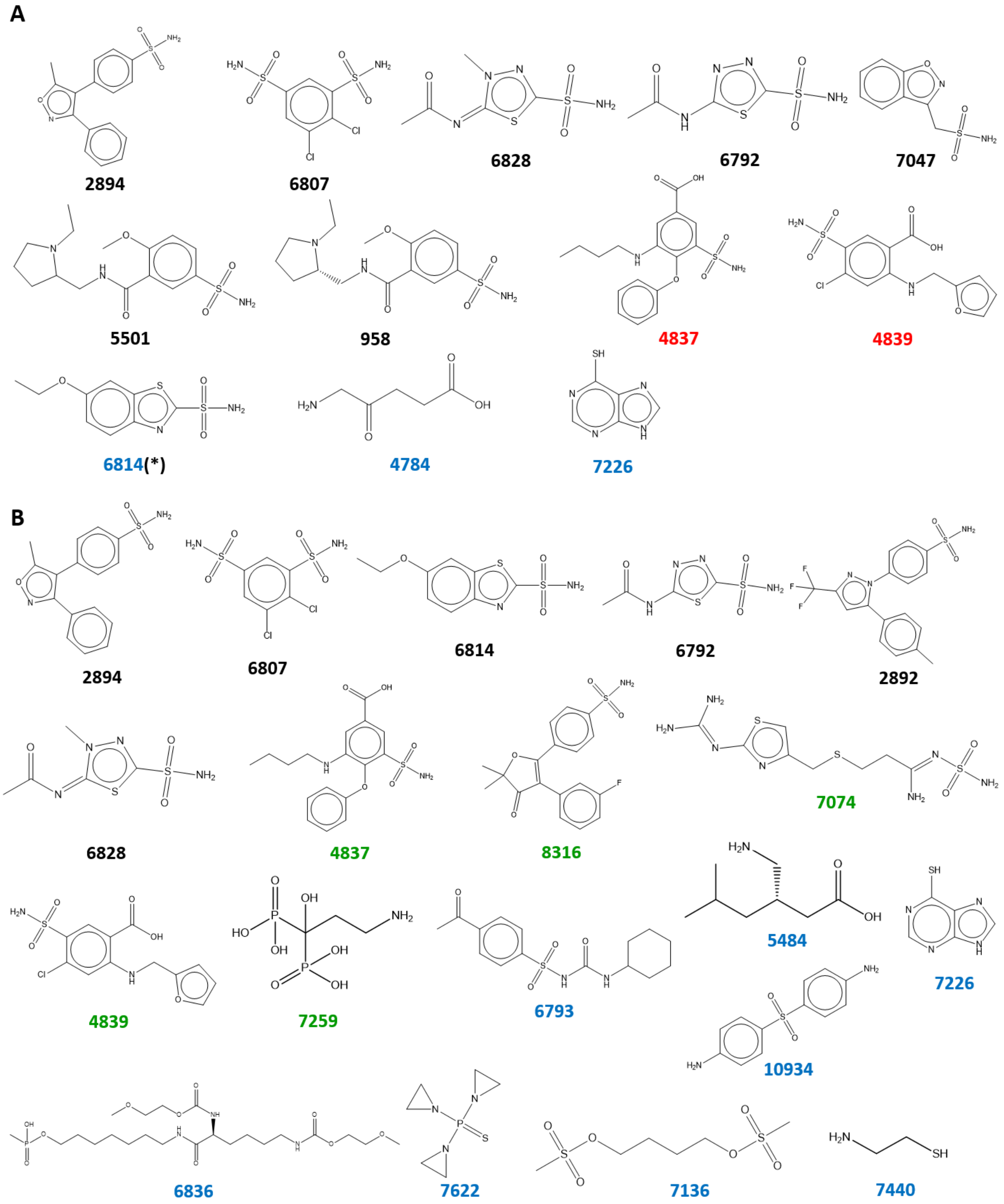

Figure S1. CA inhibitor candidates of FDA-approved small molecules by ML classifiers. (**A**) Inhibitor candidates predicted by ML classifiers (Logit + ECFP4 & MLP + ECFP4) derived from CA I inhibitors. Corresponding IUPHAR IDs are labeled [1]. The IDs colored in black are the true positives common in both ML models. Those in red are the false positives in both ML models. Those in blue except for **6814** are the false positives from MLP + ECFP4. **6814** is the true positive by MLP + ECFP4. (**B**) Inhibitor candidates predicted by ML classifiers (Logit + ECFP4 & MLP + ECFP4) derived from CA II inhibitors. The IDs in black are the true positives common in both ML models. Those in green and blue are the false positives from Logit + ECFP4 and MLP + ECFP4, respectively.

A

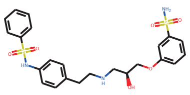

3463

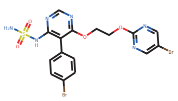

10070

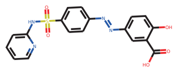

4840

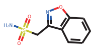

7047

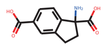

1376

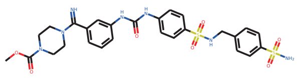

10175

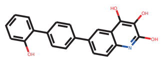

8821

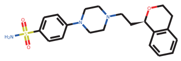

980

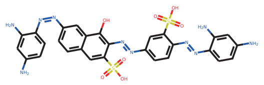

9092

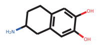

932

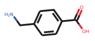

4702

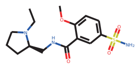

960

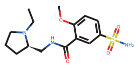

958

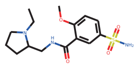

5501

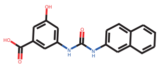

10321

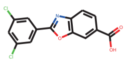

8378

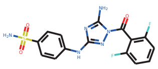

5932

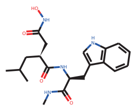

7409

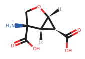

1394

B

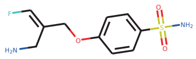

9753

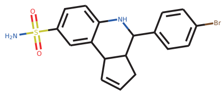

3962

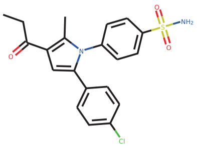

3986

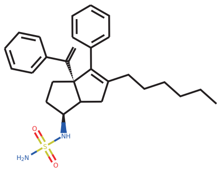

10483

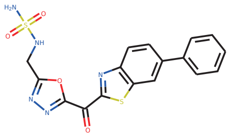

10664

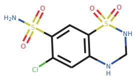

4836

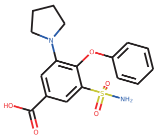

4742

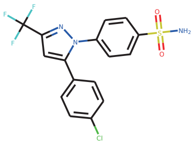

10242

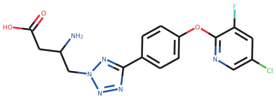

11205

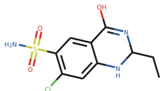

7289

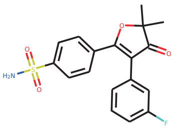

8316

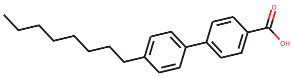

4055

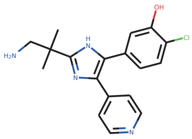

11201

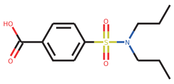

4357

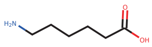

6574

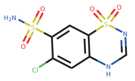

4835

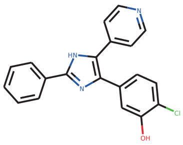

9129

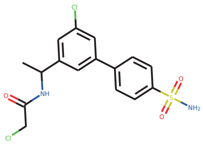

11174

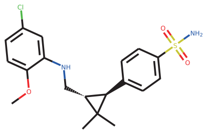

10693

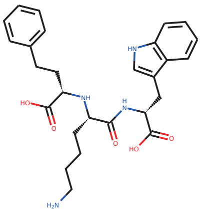

7893

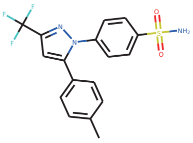

2892

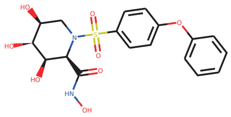

6514

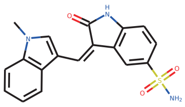

6046

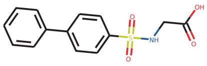

7870

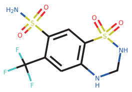

7197

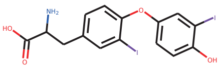

6648

C

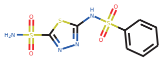

6920

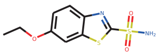

6814

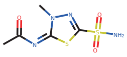

6828

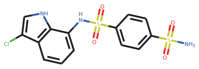

7046

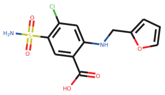

4839

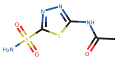

6792

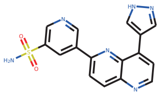

10017

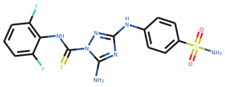

5946

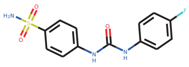

10149

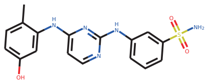

8146

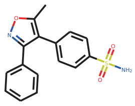

2894

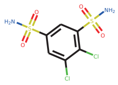

6807

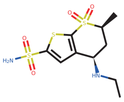

6810

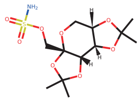

6849

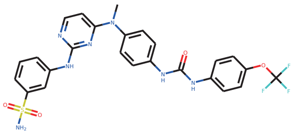

9513

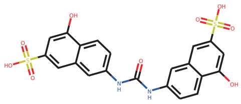

7028

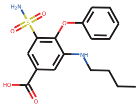

4837

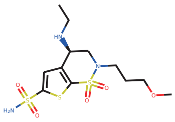

6797

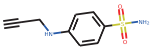

10433

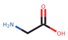

4635

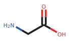

4084

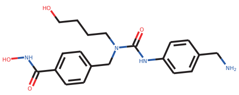

11220

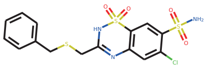

7125

Figure S2. CA inhibitor candidates from IUPHAR-registered small molecules by ML classifiers. (**A**) Inhibitor candidates predicted only by ML model (Logit + ECFP4) derived from CA I inhibitors. (**B**) Inhibitor candidates predicted only by ML model (Logit + ECFP4) derived from CA II. (**C**) Inhibitor candidates predicted both by ML models (Logit + ECFP4) derived from CA I and CA II inhibitors.

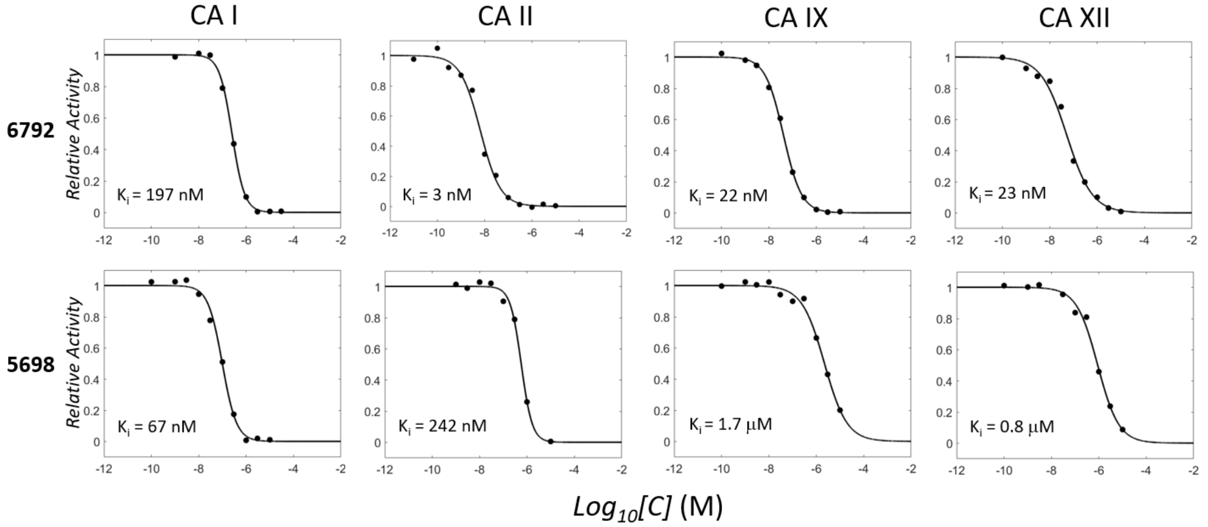

Figure S3. Concentration-dependent inhibitory profiles of positive controls. (**A**) **6792** (acetazolamide). (**B**) **5698** (pazopanib). Enzyme activities are scaled to have a relative value in the range of 0–1 on the y-axis. The enzyme activity in the absence of inhibitor is expressed as 1. The molar concentrations in each small molecule ([*C*]) are expressed as $Log_{10}[C]$ on the x-axis. The K_i values that are converted from the fitted IC_{50} values are written with units.

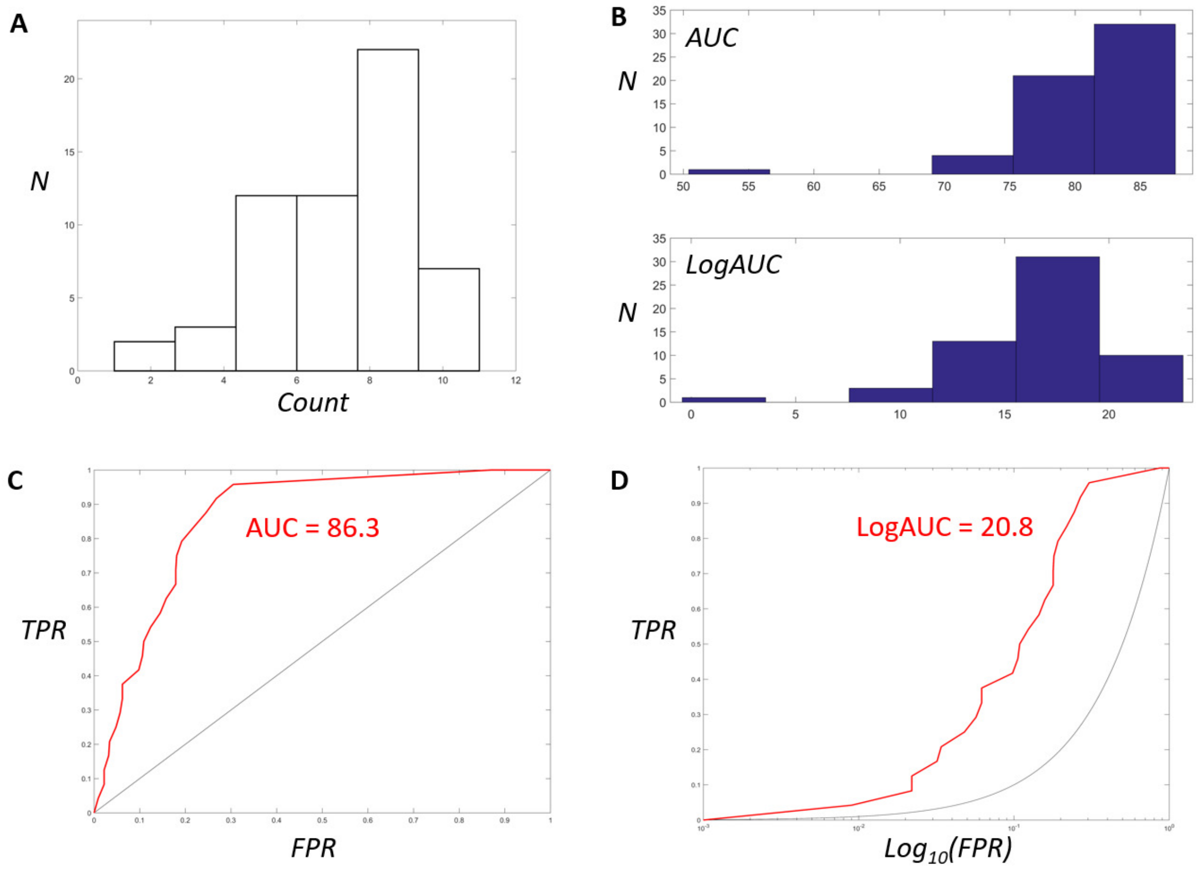

Figure S4. Data in the pre-docking stage. (**A**) Histogram of the counts in pose reproducibility. Twenty-four crystal ligands were docked into 58 coordinates from 33 CA I crystal structures using Glide-SP software [2,3]. Docked ones within 2 Å root mean square deviation into the crystal ligands are counted as the correct reproducibility. (**B**) Histograms of AUC and LogAUC. The 24 ligands and their corresponding 960 decoys (40 per ligand) were docked into 58 coordinates using Glide-SP. The AUC and LogAUC [4] values in each coordinate are calculated and expressed as histograms. Both AUC and LogAUC represent the enrichments of true positives over false positives. LogAUC has the logarithmically scaled x-axis compared to AUC in the range of 0.001–1. LogAUC value is adjusted and normalized to reflect only the enrichment of true positives by subtracting the random enrichment (0.145) and dividing by 3. Both AUC and LogAUC are multiplied by 100. Profiles of AUC (**C**) and LogAUC (**D**) in the docking with 4WUP Chain B. TPR and FPR mean true positive rate and false positive rate, respectively.

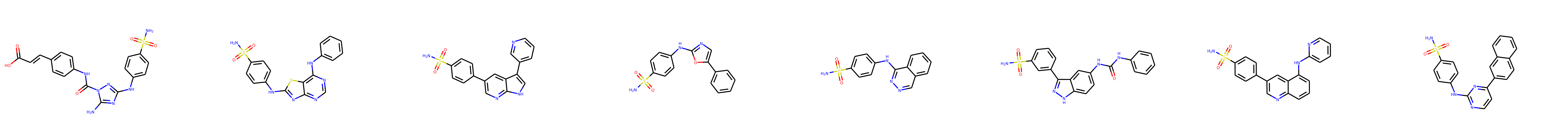
CHEMBL4525245(0.997) CHEMBL511839(0.995) CHEMBL4164814(0.992) CHEMBL194062(0.989) CHEMBL3926223(0.988) CHEMBL3330259(0.988) CHEMBL400863(0.988) CHEMBL569046(0.987)

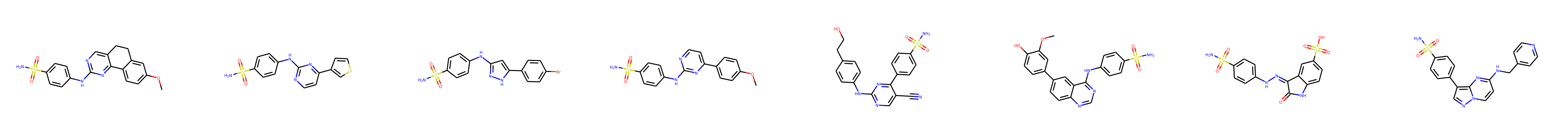
CHEMBL1082512(0.987) CHEMBL1084970(0.986) CHEMBL319903(0.985) CHEMBL1082511(0.985) CHEMBL234275(0.985) CHEMBL3661230(0.984) CHEMBL412091(0.983) CHEMBL4536438(0.982)

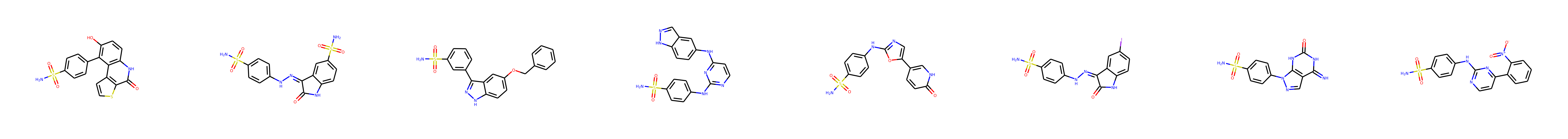
CHEMBL3667203(0.981) CHEMBL272387(0.980) CHEMBL3330253(0.979) CHEMBL3818193(0.979) CHEMBL1271692(0.978) CHEMBL273060(0.977) CHEMBL4161364(0.977) CHEMBL3924516(0.976)

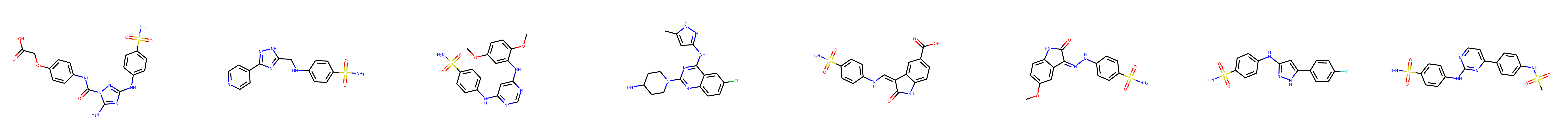
CHEMBL4476380(0.976) CHEMBL4100891(0.976) CHEMBL4276965(0.976) CHEMBL409104(0.974) CHEMBL272668(0.973) CHEMBL260103(0.970) CHEMBL103382(0.969) CHEMBL585916(0.968)

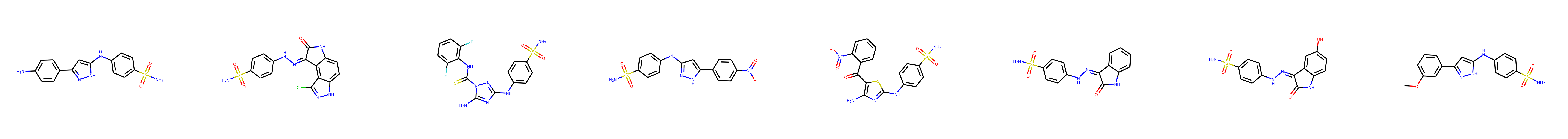
CHEMBL321517(0.967) CHEMBL269882(0.967) CHEMBL261720(0.966) CHEMBL317703(0.966) CHEMBL2377825(0.965) CHEMBL438721(0.965) CHEMBL409690(0.965) CHEMBL319685(0.964)

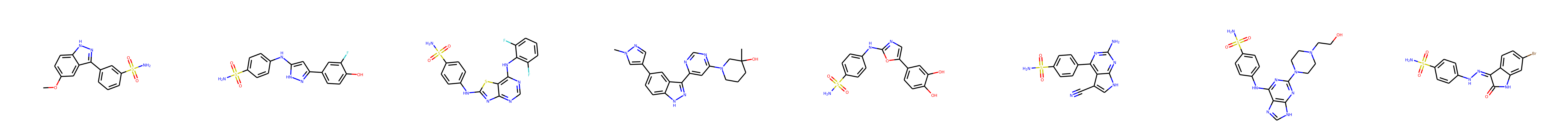
CHEMBL3330247(0.963) CHEMBL103115(0.963) CHEMBL513716(0.963) CHEMBL398469(0.962) CHEMBL1271642(0.962) CHEMBL4089551(0.962) CHEMBL1173298(0.961) CHEMBL271183(0.961)

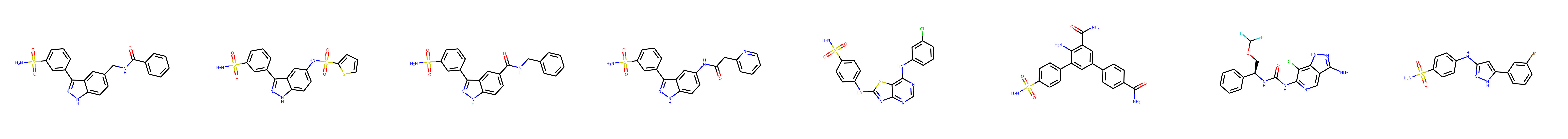
CHEMBL3330267(0.959) CHEMBL3330257(0.959) CHEMBL3330264(0.957) CHEMBL3330270(0.955) CHEMBL513418(0.955) CHEMBL514582(0.952) CHEMBL411491(0.950) CHEMBL103285(0.950)

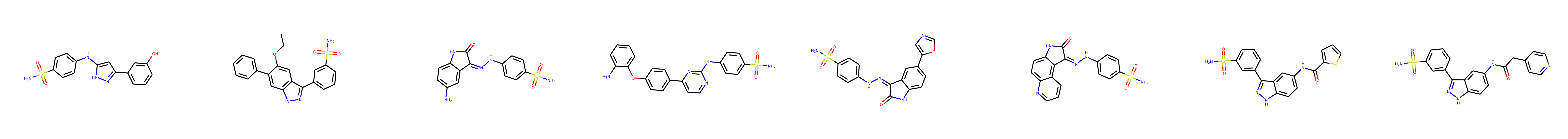
CHEMBL103104(0.950) CHEMBL2380580(0.950) CHEMBL271842(0.947) CHEMBL3942409(0.946) CHEMBL269827(0.944) CHEMBL409892(0.944) CHEMBL3330250(0.944) CHEMBL3330271(0.943)

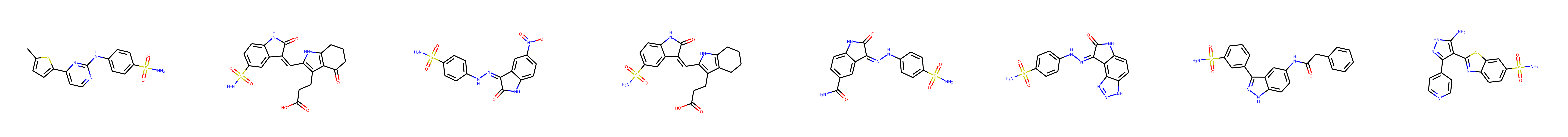
CHEMBL569998(0.942) CHEMBL1214997(0.941) CHEMBL411886(0.939) CHEMBL86531(0.938) CHEMBL410883(0.937) CHEMBL271952(0.933) CHEMBL3330255(0.930) CHEMBL3685631(0.930)

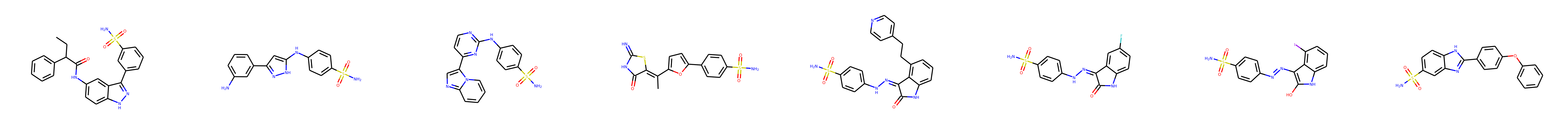
CHEMBL3330279(0.927) CHEMBL319831(0.925) CHEMBL73303(0.924) CHEMBL231950(0.923) CHEMBL1794054(0.921) CHEMBL273061(0.920) CHEMBL314397(0.920) CHEMBL176015(0.919)

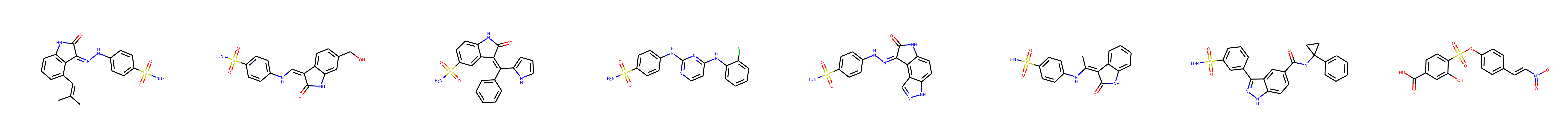
CHEMBL270544(0.917) CHEMBL410293(0.916) CHEMBL267837(0.915) CHEMBL2170410(0.915) CHEMBL272833(0.913) CHEMBL405042(0.909) CHEMBL3330278(0.907) CHEMBL77688(0.907)

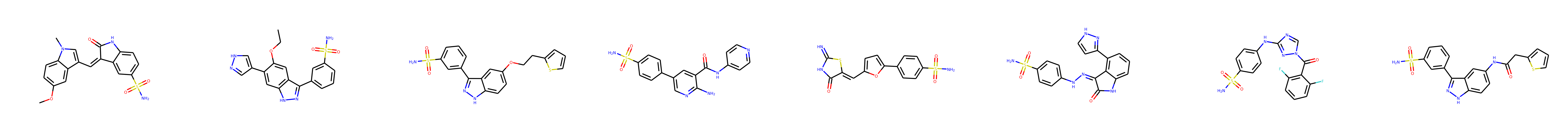
CHEMBL104333(0.904) CHEMBL2380581(0.903) CHEMBL3330254(0.898) CHEMBL3915353(0.896) CHEMBL233149(0.893) CHEMBL272684(0.891) CHEMBL4474459(0.890) CHEMBL3330256(0.890)

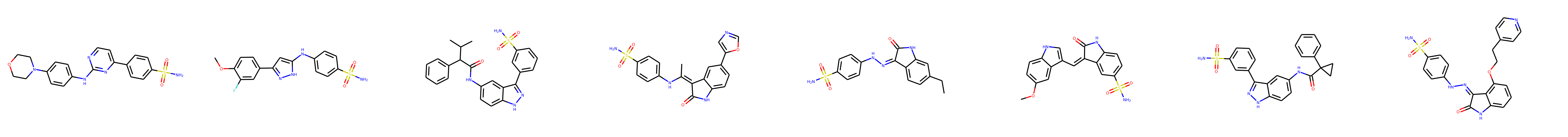
CHEMBL1080954(0.888) CHEMBL102241(0.884) CHEMBL3330280(0.884) CHEMBL411887(0.882) CHEMBL271182(0.882) CHEMBL106226(0.881) CHEMBL3330277(0.880) CHEMBL272217(0.880)

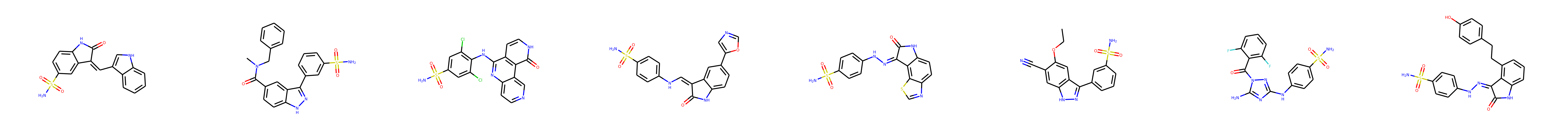
CHEMBL321955(0.878) CHEMBL3330266(0.878) CHEMBL3126350(0.875) CHEMBL270132(0.875) CHEMBL407709(0.874) CHEMBL2380578(0.872) CHEMBL191003(0.870) CHEMBL411865(0.869)

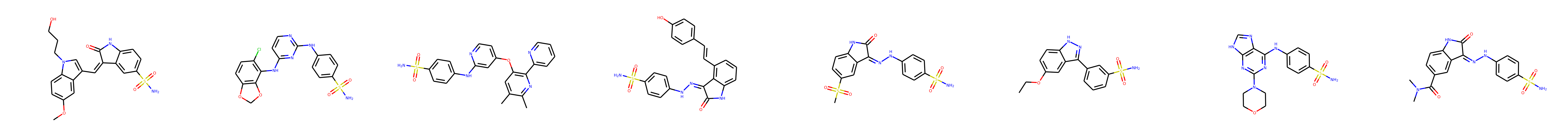
CHEMBL107061(0.869) CHEMBL256348(0.864) CHEMBL578235(0.861) CHEMBL272380(0.858) CHEMBL272389(0.858) CHEMBL2380577(0.856) CHEMBL1172957(0.855) CHEMBL409868(0.855)

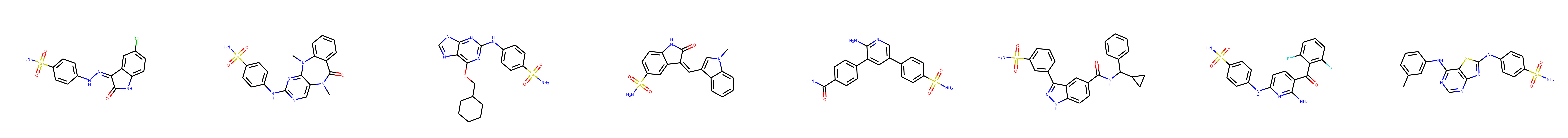
CHEMBL270054(0.851) CHEMBL1673047(0.850) CHEMBL319467(0.847) CHEMBL104279(0.847) CHEMBL4513868(0.843) CHEMBL3330281(0.842) CHEMBL193643(0.840) CHEMBL513362(0.837)

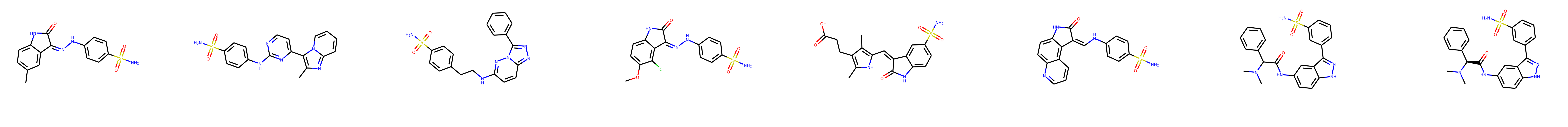

CHEMBL271843(0.836) CHEMBL72510(0.835) CHEMBL1952147(0.830) CHEMBL271822(0.829) CHEMBL150177(0.825) CHEMBL445945(0.823) CHEMBL3329232(0.822) CHEMBL3330405(0.822)

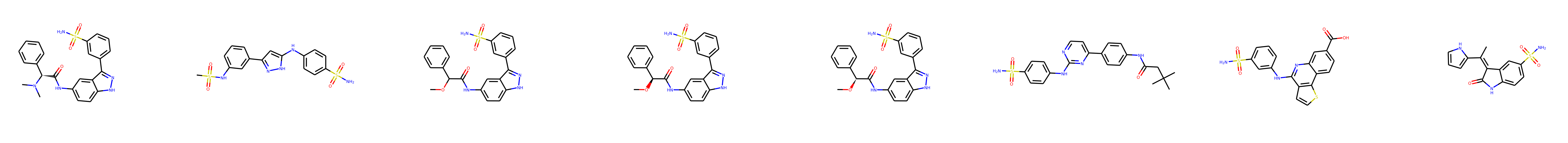

CHEMBL3330406(0.822) CHEMBL421578(0.819) CHEMBL3330401(0.819) CHEMBL3330402(0.819) CHEMBL3330403(0.819) CHEMBL1082834(0.811) CHEMBL3700519(0.805) CHEMBL234601(0.801)

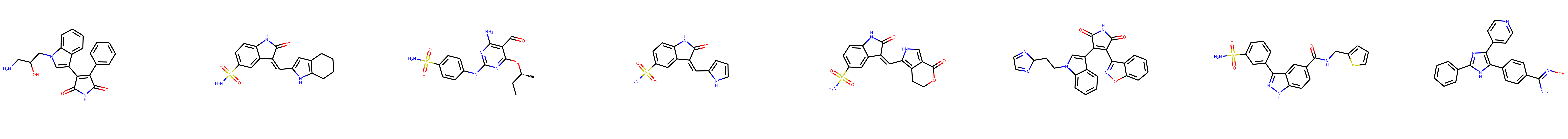

CHEMBL404077(0.801) CHEMBL88483(0.786) CHEMBL4088242(0.783) CHEMBL441911(0.781) CHEMBL432491(0.781) CHEMBL4084151(0.779) CHEMBL3330265(0.778) CHEMBL200431(0.775)

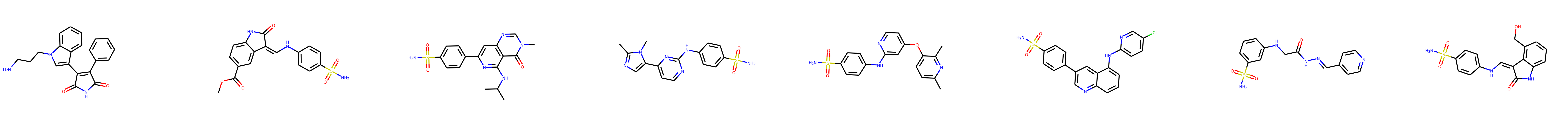

CHEMBL256827(0.772) CHEMBL409731(0.771) CHEMBL3701445(0.756) CHEMBL484738(0.753) CHEMBL585367(0.752) CHEMBL250017(0.747) CHEMBL3731114(0.745) CHEMBL271137(0.730)

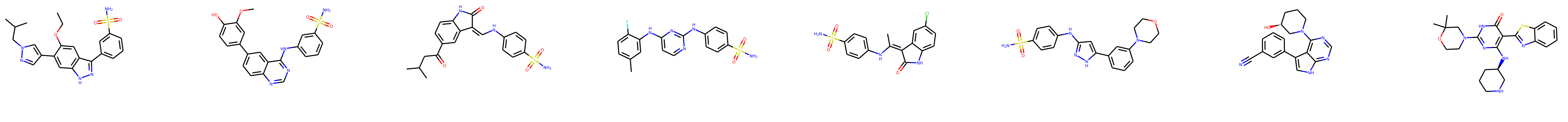

CHEMBL2380584(0.730) CHEMBL3661216(0.724) CHEMBL429142(0.719) CHEMBL103355(0.716) CHEMBL412097(0.715) CHEMBL103039(0.714) CHEMBL411224(0.710) CHEMBL411425(0.704)

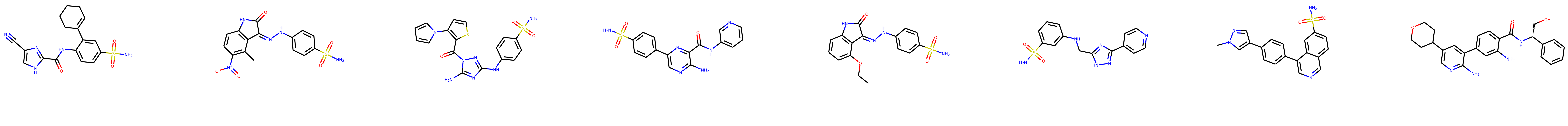

CHEMBL3086161(0.700) CHEMBL272667(0.696) CHEMBL483082(0.694) CHEMBL2177164(0.691) CHEMBL411696(0.691) CHEMBL4072686(0.690) CHEMBL3827333(0.690) CHEMBL411490(0.685)

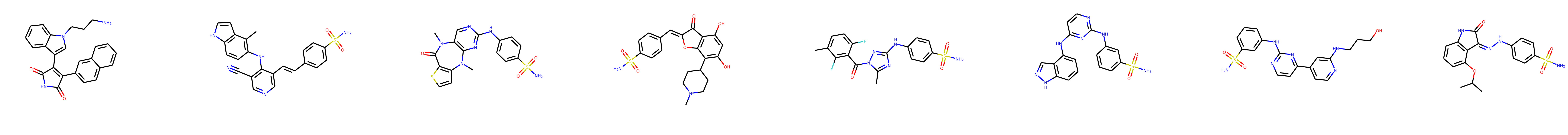

CHEMBL409834(0.680) CHEMBL1080801(0.678) CHEMBL4554938(0.674) CHEMBL48362(0.673) CHEMBL363607(0.670) CHEMBL398894(0.667) CHEMBL2158843(0.661) CHEMBL271160(0.654)

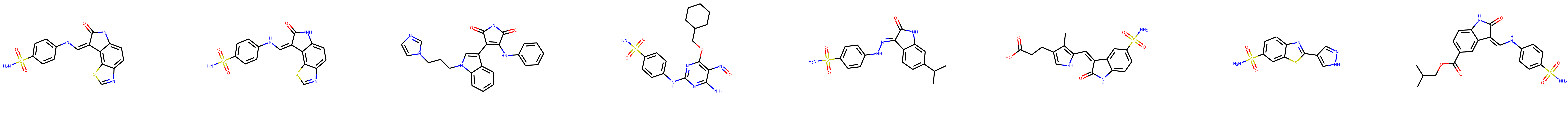

CHEMBL1794055(0.634) CHEMBL271595(0.634) CHEMBL366266(0.630) CHEMBL318999(0.626) CHEMBL271594(0.619) CHEMBL146697(0.608) CHEMBL3680992(0.604) CHEMBL269883(0.600)

Figure S5. ML-derived CA inhibitor candidates of known kinase inhibitors. The ChEMBL-deposited [5] 35,455 kinase inhibitors from 437 kinases were extracted. The molecules smaller than 450 Da and more potent than 500 nM in K_i or IC_{50} were considered. The ML classifier trained with CA I inhibitors in this study generated 192 candidates with $p \geq 0.6$. The values in the parentheses are probability by the ML classifier.

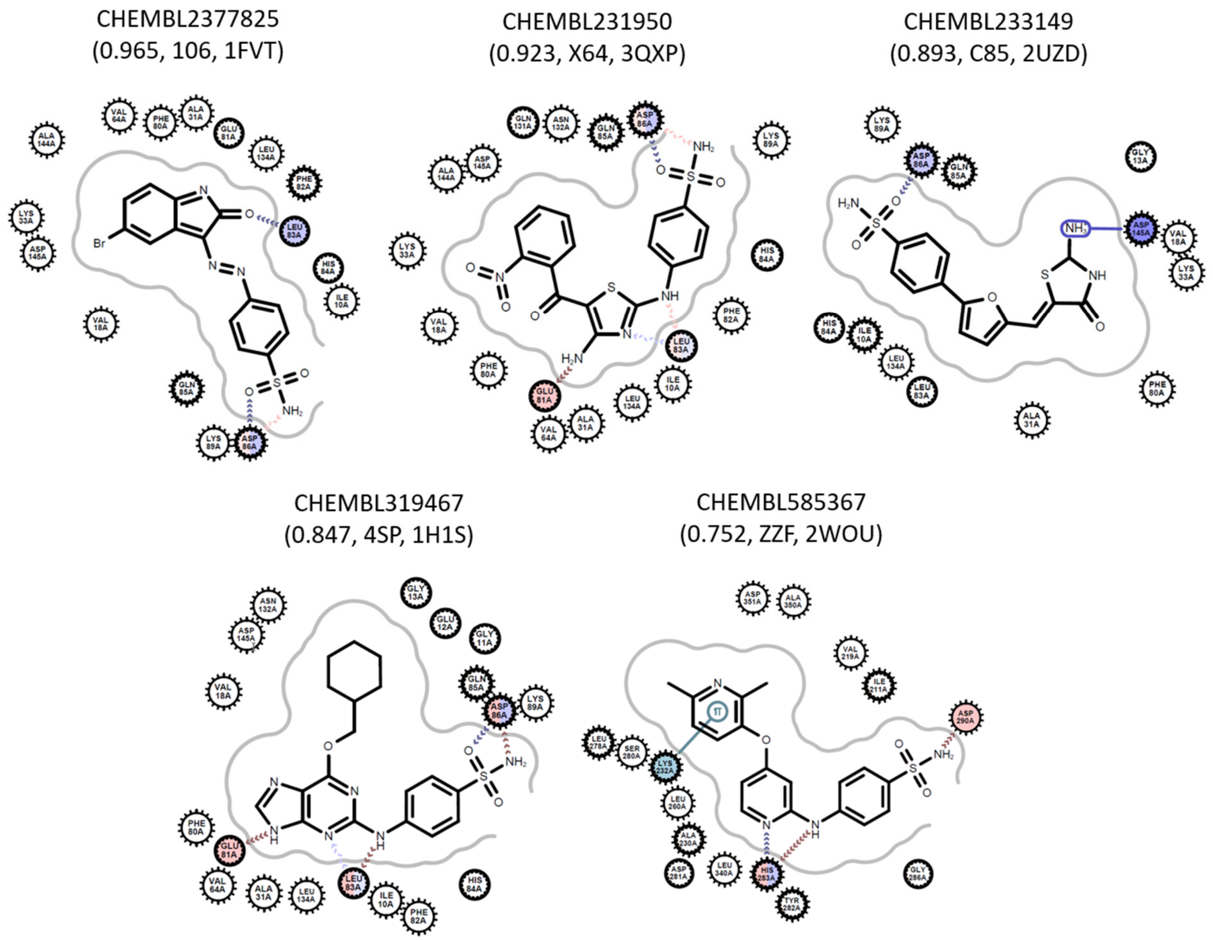

Figure S6. CA inhibitor candidates reported as complex structures with kinases. The 192 kinase inhibitors chosen as CA inhibitor candidates include eight molecules reported as complex structures with kinases. The values in the parentheses are probability by ML classifier, name of the ligand, and PDB ID of the kinase structure. The identical definition to Figure 5 is applied for representing the intermolecular interactions. Besides the five molecules in this figure, the other three are **5932** and **5946** (Figure 5) and XMU-MP-1 (Figure 6).

References

1. Armstrong, J.F.; Faccenda, E.; Harding, S.D.; Pawson, A.J.; Southan, C.; Sharman, J.L.; Campo, B.; Cavanagh, D.R.; Alexander, S.P.H.; Davenport, A.P., et al. The IUPHAR/BPS Guide to PHARMACOLOGY in 2020: extending immunopharmacology content and introducing the IUPHAR/MMV Guide to MALARIA PHARMACOLOGY. *Nucleic acids research* **2020**, *48*, D1006-D1021, doi:10.1093/nar/gkz951.
2. Halgren, T.A.; Murphy, R.B.; Friesner, R.A.; Beard, H.S.; Frye, L.L.; Pollard, W.T.; Banks, J.L. Glide: a new approach for rapid, accurate docking and scoring. 2. Enrichment factors in database screening. *Journal of medicinal chemistry* **2004**, *47*, 1750-1759, doi:10.1021/jm030644s.
3. Friesner, R.A.; Banks, J.L.; Murphy, R.B.; Halgren, T.A.; Klicic, J.J.; Mainz, D.T.; Repasky, M.P.; Knoll, E.H.; Shelley, M.; Perry, J.K., et al. Glide: a new approach for rapid, accurate docking and scoring. 1. Method and assessment of docking accuracy. *Journal of medicinal chemistry* **2004**, *47*, 1739-1749, doi:10.1021/jm0306430.
4. Mysinger, M.M.; Shoichet, B.K. Rapid context-dependent ligand desolvation in molecular docking. *Journal of chemical information and modeling* **2010**, *50*, 1561-1573, doi:10.1021/ci100214a.
5. Gaulton, A.; Hersey, A.; Nowotka, M.; Bento, A.P.; Chambers, J.; Mendez, D.; Mutowo, P.; Atkinson, F.; Bellis, L.J.; Cibrian-Uhalte, E., et al. The ChEMBL database in 2017. *Nucleic acids research* **2017**, *45*, D945-D954, doi:10.1093/nar/gkw1074.
